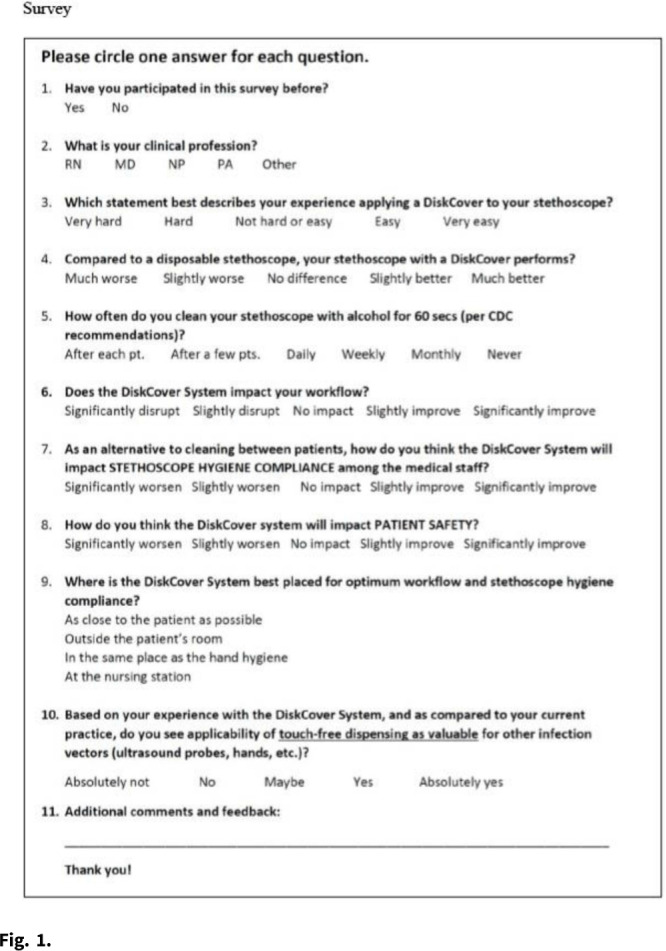# Stethoscope hygiene, workflow, and patient safety: The crux of healthcare-associated infections

**DOI:** 10.1017/ash.2022.162

**Published:** 2022-05-16

**Authors:** William Peacock, Stuart Kipper, Sean-Xavier Neath

## Abstract

**Objective:** We evaluated the impressions and perceived workflow consequences following installation of a touch-free aseptic stethoscope barrier dispenser in the clinical environment. **Methods:** Beginning in 2020, we conducted a volunteer survey of aseptic stethoscope diaphragm barrier (AseptiScope, San Diego, CA) users in multiple departments at 7 US healthcare facilities. A 10-question survey was presented on an iPad near the aseptic barrier dispenser, which was usually located in the patient exam room, to be available immediately after the practitioner completed their examination, which included the use of the stethoscope barrier. This evaluation was considered a quality improvement project and was exempt from institutional review board approval. For this analysis, only 1 survey per practitioner was included. **Results:** Overall, 147 surveys were obtained from 7 institutions geographically distributed across the United States, immediately after placement of the DiskCover system in the patient care environment. Responses were generally positive and included ease of use (95.2% rated easy or very easy), comparison to a disposable stethoscope (97.9% as similar to, improved over, or significant improvement), workflow changes (53.7% improvement, 97.3% no impact, or improved), and perceived effect on patient safety (90.3% felt that patient safety was improved or significantly improved). **Conclusions:** The use of a touch-free aseptic stethoscope barrier system was reported to be easy to use, superior to a disposable stethoscope, and an improvement to practitioner workflow and perceived patient safety.

**Funding:** AseptiScope, Inc.

**Disclosures:** None

**Figure:**
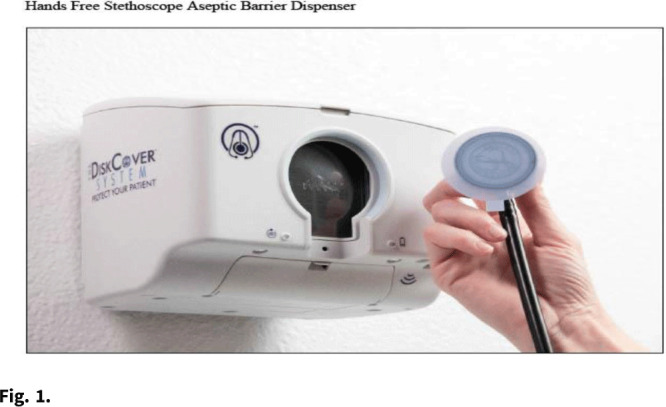

**Figure:**